# Ironing out Macrophage Immunometabolism

**DOI:** 10.3390/ph12020094

**Published:** 2019-06-19

**Authors:** Stefania Recalcati, Elena Gammella, Gaetano Cairo

**Affiliations:** Department of Biomedical Sciences for Health, University of Milan, 20133 Milan, Italy; stefania.recalcati@unimi.it (S.R.); elena.gammella@unimi.it (E.G.)

**Keywords:** macrophages, iron, metabolism, inflammation

## Abstract

Over the last decade, increasing evidence has reinforced the key role of metabolic reprogramming in macrophage activation. In addition to supporting the specific immune response of different subsets of macrophages, intracellular metabolic pathways also directly control the specialized effector functions of immune cells. In this context, iron metabolism has been recognized as an important component of macrophage plasticity. Since macrophages control the availability of this essential metal, changes in the expression of genes coding for the major proteins of iron metabolism may result in different iron availability for the macrophage itself and for other cells in the microenvironment. In this review, we discuss how macrophage iron can also play a role in immunometabolism.

## 1. Introduction

Over the last decade, interest in immunometabolism—the interaction between immunological and metabolic systems at both the body and cellular levels—has grown considerably. Although the metabolic pathways of several types of immune cells, such as T cells—which contribute to immune modulation in cancer through their metabolic connection with tumor cells [1]—have received attention, most studies have focused on macrophage immunometabolism. In fact, it is increasingly appreciated that the functions of these cells extend beyond immune defense. Indeed, metabolism is not only a system to produce energy, but also a source of a variety of intermediates with relevant biological function in innate immunity and inflammatory response. Under the influence of signals from the microenvironment, cells of the monocyte–macrophage lineage undergo a complex reprogramming toward distinct functional phenotypes [2]. Emerging evidence has shown that immunometabolism is an important factor of the functional phenotype that characterizes the different macrophage populations, such as those present in sites of infections, tissue injury, tumors, atherosclerotic plaques, adipose tissue, etc. In this review, we address the cell-intrinsic metabolic functions that contribute to determine the functional activity of macrophages focusing in particular on iron metabolism, which has been recognized as one of the selected metabolic features of different classes of macrophages [3].

## 2. Macrophage Polarization

**Figure 1:**
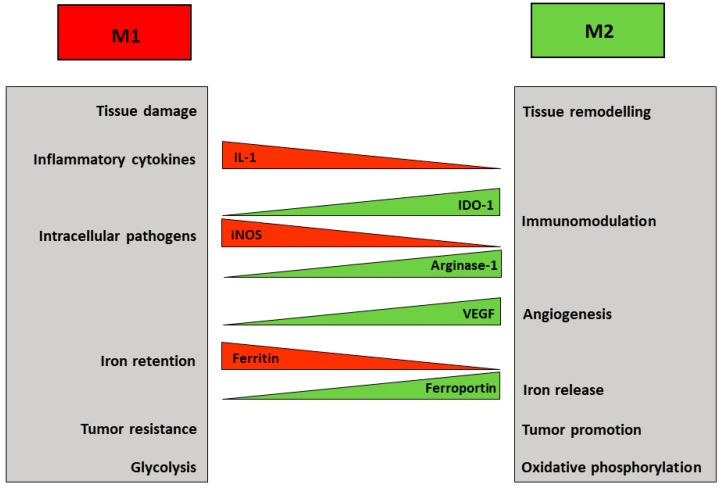
M1 and M2 macrophages represent the extremes of a spectrum. The major properties and functions of polarized macrophages are summarized in the boxes. The differential expression of representative molecules and effectors is also shown.

Macrophages play a variety of distinct roles both in pathological conditions, such as inflammation, and physiological tissue homeostasis. To fulfil these tasks, macrophage populations are able to acquire different phenotypes depending on environmental and immune signals, which polarize macrophages to a range of activation states classified in two major groups: classical pro-inflammatory (M1) macrophages endowed with cytotoxic and microbicidal functions, which are typically induced following activation of toll-like receptors (TLR) by pathogen products such as lipopolysaccharide (LPS), or alternative anti-inflammatory (M2) macrophages, which respond to anti-inflammatory cytokines like interleukin 4 (IL-4) and are involved in cell growth and tissue remodelling [4] (Figure 1). In spite of the oversimplification, this classification faithfully represents the polarization of macrophages in most pathophysiological settings. The behaviour and the gene expression profile of differently polarized macrophages show considerable differences, but recent studies have shown that several divergences in cellular metabolism also contribute significantly to the diversity of the M1 and M2 populations. Notably, the metabolic changes, which are influenced by signals from the microenvironment, in turn support the specialized functions of macrophage subsets [5]. In addition, recent studies showed that altering macrophage metabolism can also reshape their functions [6]. This may offer the possibility to exploit metabolic pathways to alter the role of macrophages in a variety of pathologic conditions in which myelomonocytic cells are a key component, such as tumors, obesity, atherosclerosis.

## 3. Macrophage Diversity and Metabolism 

### 3.1. Amino Acid Metabolism

Macrophage plasticity includes changes in amino acid metabolism (Figure 1); in fact, a well-known distinctive feature of M1 macrophages is the inducible conversion of arginine into nitric oxide (NO), a reactive compound important for microbial killing. Conversely, arginase-dependent metabolism of arginine in M2 cells on the one hand depletes arginine required for NO synthesis in macrophages and for T cell immunity [7]; on the other hand leads to the production of ornithine, thus possibly contributing to collagen synthesis, consistent with the healing function of M2 [3,5].

**Figure 2:**
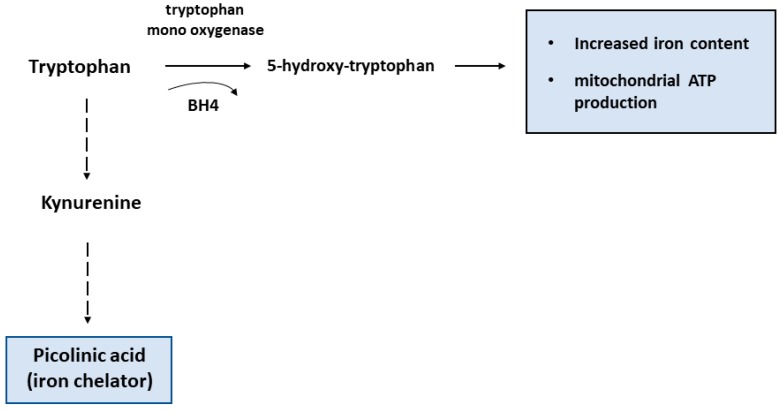
Schematic diagram of different tryptophan metabolism pathways. According to results found in T cells lacking tetrahydrobiopterin (BH4) (see text), it is conceivable that the conversion of tryptophan by tryptophan mono-oxygenase results in high iron levels and efficient mitochondrial activity, whereas the kynurenine pathway initiated by IDO1 activity leads to the formation of the iron chelator picolinic acid.

The metabolism of tryptophan which can be converted in different compounds along two main pathways represents another example of the connection between immune function and amino acid metabolism. Tryptophan can be metabolized by the heme-containing indoleamine 2,3-dioxygenase 1 (IDO1) or tryptophan mono-oxygenase: IDO1 catalyzes the initial reaction of the pathway that transforms tryptophan into kynurenine, a product that exerts major immunosuppressive effects by inhibiting T cell immunity and impairing chemotaxis of neutrophils [7] (Figure 2). IDO1 can be induced in several types of myeloid cells, including macrophages [8], in response to stimulation of innate immunity [9]. The immunosuppressive activity of IDO1, which is also considered a marker of M2 polarization, has been ascribed to its ability to deplete the microenvironmental tryptophan pools for T cells while favoring the accumulation of kynurenine and the production of ligands for the aryl hydrocarbon receptor with ensuing synthesis of pro-inflammatory cytokines [10]. Consistent with the inhibitory role of kynurenine, lack of tetrahydrobiopterine (BH4), which is a cofactor for tryptophan mono-oxygenase, favored tryptophan metabolism by IDO1, thus repressing T cells proliferation and impairing mitochondrial respiration and energy production [11].

### 3.2. Glucose Metabolism

**Figure 3:**
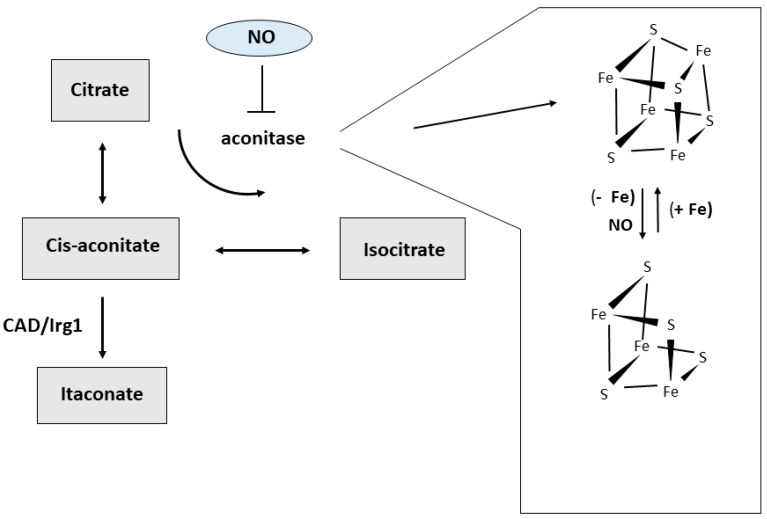
Alternative pathways of cis-aconitate metabolism. Iron availability and reactive molecules produced under inflammatory condition like nitric oxide (NO) can target the iron-sulfur cluster and alter aconitase activity, thus affecting the amount of cis-aconitate available for the decarboxylating activity of CAD/Irg1 and the production of itaconate.

The differences in amino acid metabolism described above are well-known and have long been used to define macrophages subsets, but recently a growing interest highlighted the role of glucose metabolism in this context (reviewed in Reference [12,13,14]) (Figure 1). M1 macrophages produce ATP mainly through aerobic glycolysis, i.e., the metabolism of glucose to lactate despite oxygen availability. Reactive oxygen species-mediated hypoxia inducible factor 1 α (HIF1α) induction results in glycolysis upregulation [15]. This pathway is less efficient in generating ATP than the mitochondrial respiration, but it allows the cells to generate building blocks needed for the synthesis of nucleotides, amino acids and lipids. Moreover, the higher levels of glucose 6-phosphate generated by increased glycolysis can stimulate the pentose phosphate pathway, thus also providing more of the reduced form of nicotinamide adenine dinucleotide phosphate (NADPH), a key cofactor for a variety of biosynthetic processes, including lipid synthesis. Therefore, M1 macrophages adapt their energy metabolism to secure enough ATP, while also supporting biosynthetic functions needed to sustain proliferation and synthesis of cell structures and inflammatory molecules. At the same time, the other metabolic process observed in M1 macrophages is the inhibition of mitochondrial oxidative phosphorylation [12]. This is attained by suppressing the tricarboxylic acid (TCA) cycle at two distinct levels, with concomitant generation of intermediates that are used for protein and lipid synthesis. The block of isocitrate dehydrogenase (IDH) results in the accumulation of acetyl CoA and citrate, which is then exported from the mitochondria to the cytosol where it can be converted by acetyl-CoAcarboxylase (ACC) to malonyl-CoA, which is then used to synthesize fatty acids, important precursors for the expansion of membranes, such as the endoplasmic reticulum. Suppression of succinate dehydrogenase (SDH) is obtained through a recently discovered pathway based on TLR4-mediated induction of *cis*-aconitic acid decarboxylase/immune-responsive gene 1 (CAD/Irg1), an enzyme that drives the conversion of cis-aconitate, the intermediate between citrate and isocitrate in the TCA cycle, to itaconate, a metabolite possessing both anti-inflammatory and anti-microbial properties [16] (Figure 3). It has been shown that itaconate, known as industrial polymer since almost a century [17], inhibits SDH activity, thereby decreasing fumarate production. Since SDH is also a component of the complex II of the electron transport chain, induction of CAD/Irg1 activity affects two key steps of cellular oxidative energy production. Moreover, by inhibiting the prolyl hydroxylases involved in HIF1α degradation pathway, succinate accumulation may concur to the induction of glycolysis reported above. The latter effect is also achieved by itaconate-mediated repression of fatty acid biosynthesis from glucose. Interestingly, itaconate is also endowed with antimicrobial properties against a variety of microorganisms, as it is transported from the mitochondria, where CAD/Irg1 is located, to phagosomes in which it inhibits microbial isocitrate lyase, an enzyme involved in the glyoxylate pathway required for survival when glucose availability is limited [16]. Itaconate is also secreted [17], as revealed by its presence in serum, and hence it also traverses the cytoplasm. Therefore, mitochondrial or cytosolic itaconate might play regulatory roles while phagosomal and extracellular itaconate participates in anti-microbial action. A recent study [18] reported that the activity of one isoform (BCAT1) of branched chain aminotransferases, multi-level regulators of the TCA cycle and oxidative phosphorylation, is required for itaconate production and metabolic reprogramming in human macrophages, thus showing the interaction of different nutrient metabolism (amino acids and glucose) in pro-inflammatory macrophage function.

Notably, the link between macrophage metabolism and functionality is underlined by the evidence that metabolic pathways of M1 macrophages contribute specifically to their pro-inflammatory role. Moreover, metabolites can also be employed as immunoregulators in order to control the extent and duration of the inflammatory response. For example, the lactate produced by activated M1 macrophages through glycolysis accumulate and exerts an immunosuppressive function by stimulating M2 polarization of macrophages. Interestingly, lactate appears to be one of the factors produced by tumor cells that promote the formation of M2-like tumor associated macrophages (TAM) [19].

On the other hand, M2 cells show high levels of oxidative phosphorylation and mitochondrial activity. The TCA cycle is fueled by consumption of fatty acids undergoing beta oxidation and also by glutamine utilization to form α-ketoglutarate. To support these reactions, mitochondrial biogenesis is also induced. Interestingly, the key metabolic regulator mechanistic target of rapamycin (mTOR) is involved also in macrophage metabolism and activation. In fact, inducers of M2 polarization like IL-4 signal through the mTOR pathway to promote fatty acid oxidation (FAO) and glucose consumption for oxidative phosphorylation, two metabolic changes that are critical for macrophage alternative activation [20]. A recent study has demonstrated that upregulating FAO in macrophages of hypercholesterolemic mice via miR-33 inhibition drives macrophages toward an M2 state and reduces atherosclerosis [21]. This study emphasizes how metabolic reprogramming of macrophages can influence disease outcome.

The anti-inflammatory and pro-resolving function of M2 macrophages is involved also in efferocytosis, the process by which apoptotic cells are cleared by phagocytes, primarily resident macrophages. Recent findings have shown that two distinct metabolic pathways are required to prevent unwanted inflammation and promote repair by creating an anti-inflammatory tissue environment. One study showed the importance of glycolysis and lactate release [22], whereas Zhang et al. [23] described how FAO starts a mechanism leading to the production of anti-inflammatory IL-10. These results indicate that macrophages adopt separate but converging mechanisms to alter their metabolism and thus drive an anti-inflammatory response.

Amino acid and glucose metabolism are linked in reprogramming macrophage function. In fact, recent findings have shown that NO also impacts on mitochondrial cell bioenergetic pathways, probably thanks to its reactivity against iron-containing Fe-S clusters and heme moieties that are present in several proteins of the electron transport chain and TCA cycle. In fact, knock down of iNOS restored mitochondrial ATP production through oxidative metabolism in M1 macrophages. These forces activated macrophages to bring in glucose and use glycolytic metabolism to produce energy [12]. Notably, both the accumulation of citrate described above and the generation of glucose 6-phosphate by glycolysis, with consequent stimulation of the pentose phosphate pathway, favor NO production, thereby inducing a feedforward loop of inflammatory activation.

Despite our increased understanding of macrophage immunometabolism as an important determinant of the pathophysiological conditions in which macrophages are involved, it should be kept in mind that several issues remain to be explored and clarified. Some of the major challenges and concerns are reported below. In the same way as the distinction of M1 and M2 macrophages between pro- and anti-inflammatory, respectively, is an oversimplification, also describing their metabolism as glycolytic or depending on oxidative phosphorylation does not reflect the variety of phenotypes and metabolic profiles that can be obtained by in vitro exposure to different stimuli or, even more, to the distinct macrophage subsets occurring in vivo. An example of the diverse effects on metabolism of different M1 polarizing stimuli is represented by the response of mitochondrial metabolism to LPS, which is downregulated in bone marrow-derived macrophages and upregulated in peritoneal macrophages, despite a similarly strong inflammatory response [24]. Another striking example is provided by TAM, which are usually considered M2-like but show a glycolytic metabolism similar to that of in vitro polarized M1 cells (discussed in Reference [14]). Moreover, IRG1-itaconic acid production, which is typical of M1 cells, has been shown to be linked to the modulation of M2-like macrophage polarization in the revascularization of ischaemic muscle [25]. It should be also kept in mind that most studies investigating immunometabolism were performed in mouse models and in several instances the maintenance of the observed effects in humans remains to be investigated and demonstrated. Indeed, several differences exist between mouse and human macrophages, such as the inability of human macrophages to produce significant amounts of a key regulator of metabolic reprogramming like NO.

## 4. Macrophages and Iron Metabolism 

Iron is a redox-active metal required for the activity of essential enzymes involved in a variety of cellular functions, but excess iron is potentially toxic as a catalyst of oxidative stress. Therefore, the appropriate iron concentration in the cells and organisms is homeostatically maintained by several mechanisms. The role of macrophages in iron metabolism has been covered extensively in this Special Issue [26]. At the systemic level, macrophages are key regulators of iron trafficking [27,28]. Spi-C-dependent splenic reticuloendothelial cells clear senescent erythrocytes and release iron back to circulation, thus providing iron needed for erythroid precursors proliferation and hemoglobin synthesis [29]. On the other hand, iron sequestration by pro-inflammatory macrophages represents a recognized and efficient bacteriostatic response [30,31]. Moreover, iron is required for hemoproteins essential for macrophage activity in inflammation, such as NADPH oxidase 2, cyclooxygenases 1 and 2, inducible nitric oxide synthase, etc. [29]. Consistent with their different functions in homeostatic and inflammatory conditions, polarized macrophages also show a distinct expression of genes involved in iron homeostasis [28,32], in which M1 cells withhold iron whereas M2 are prone to iron release to tissues (Figure 1). High expression of the iron storage protein ferritin correlates with iron retention by in vitro polarized M1 macrophages. On the other hand, alternatively activated M2 macrophages show an elevated capacity for CD163-dependent uptake and heme oxygenase-mediated catabolism of heme-associated iron, as well as high expression of ferroportin (FPN), the only known mammalian iron exporter [33]. This FPN-mediated sustained iron release may significantly contribute to the role of M2 macrophages in various pathophysiological conditions, such as tissue repair, and also tumor growth [28], as TAM acquire a M2-like phenotype [34]. However, the effect of iron on TAM polarization remains to be fully understood. In fact, it has been shown that high doses of iron can shift TAM, which usually have a pro-tumor activity, to the anti-tumor M1 phenotype [35]. Conversely, it has been described that an intracellular iron-chelator can switch TAM from their iron-release phenotype toward iron storage [36]. In this context, it is interesting that TAM macrophages can also be a source of extracellular ferritin in the tumor microenvironment and ferritin was able to stimulate the growth of cancer cells in an iron-independent way, at least in vitro [37].

Moreover, we recently demonstrated that macrophage FPN-dependent supply of iron to the microenvironment is necessary for stromal and parenchymal cells multiplication, differentiation and activity, both in the physiological context of hair follicle growth and in the pathophysiological setting of wound healing, two conditions sharing many similarities including fast cell replication [38]. In fact, loss of macrophage FPN causes hair follicle alterations and alopecia by inhibiting the proliferation of neighbouring hair follicle cells. Similarly, iron retention in macrophages delayed wound healing in mice lacking FPN in macrophages by affecting stromal cells proliferation, blood and lymphatic vessels formation and fibrogenesis. These findings indicated that iron should be included in the list of trophic mediators produced by macrophages that are required for tissue homeostasis and repair. Although in these settings iron retention in macrophages had no impact on the inflammatory processes accompanying wound healing [38], other studies suggested that under conditions of massive iron deposition caused by hemolysis iron accumulation in macrophages can activate them to a pro-inflammatory M1 phenotype [39,40,41]. The conflicting results may be related to the amount of iron stored as well as to the exposure to different iron sources, as heme iron is highly toxic [42].

## 5. Macrophage Iron and Immunometabolism

The differences in iron metabolism existing in the various macrophage subsets can be relevant also for immunometabolism. As reported above, in pro-inflammatory macrophages CAD/Irg1 decarboxylates cis-aconitate to generate itaconate, a compound with antimicrobial functions [16,17]. Cis-aconitate, an intermediate in the conversion of citrate to isocitrate, is the substrate of both mitochondrial and cytosolic aconitases. The latter represents an alternative form of iron-regulatory protein 1 (IRP1), an RNA-binding protein which controls the synthesis of proteins involved in intracellular iron usage, storage or transport [43]. The availability of iron to assemble the iron-sulfur cluster dictates the switch between the enzymatically active holoprotein endowed with aconitase activity and the RNA-binding apoform which controls iron homeostasis [44] (Figure 3). We have shown that in macrophages NO is able to induce concomitant inactivation of cytosolic aconitase and activation of IRP1, attributable to disassembly of the Fe-S cluster [45]. This switch should result in cis-aconitate accumulation and increased availability of this substrate to CAD/Irg1-mediated conversion to itaconate. Therefore, this process could represent a still unexplored pathway used by NO-producing inflammatory macrophages to favor the generation of an antimicrobial compound. In addition, by targeting iron-sulfur clusters the generation of NO can also affect macrophage metabolic activities [46]. In fact, in Toll-like receptor-activated mouse macrophages, NO-mediated damage to iron-sulfur proteins compromises mitochondrial oxidative metabolism and thus constitutes an additional mechanism contributing to the shift toward the glycolytic pathway that characterizes M1 cells. Notably, genes involved in iron-sulfur cluster biogenesis, such as ISCU and NSF1, were found to be preferentially expressed in M2 polarized human macrophages [32], which rely on mitochondrial oxidative phosphorylation for energy production. In the same context, by influencing the formation of the iron-sulfur cluster of mitochondrial aconitase, iron availability in the mitochondria could determine whether cis-aconitate is decarboxylated by CAD/Irg1 or converted to isocitrate by aconitase (Figure 3).

Iron may be involved also in amino acid catabolism. Tryptophan mono-oxygenase-mediated tryptophan metabolism contributes to maintain high iron levels and efficient mitochondrial respiration [11], whereas tryptophan degradation by IDO1 through the kynurenine pathway leads to formation of picolinic acid, which is an iron chelator [47] (Figure 2). By depleting the cells of iron, which is essential for enzymes involved in DNA synthesis like ribonucleotide reductase [48], iron sequestration by picolinic acid may contribute to the immunosuppressive activity of the kynurenine pathway, which impairs T cells proliferation and activity [49]. Moreover, the ability of picolinic acid to amplify IFN-γ-mediated NO production in macrophages, an event connecting arginine and tryptophan metabolism [50], may depend on the induction of nitric oxide synthase (NOS) mRNA expression by the hypoxia inducible transcription factor (HIF), which is also induced by iron deficiency [51]. Therefore, iron may contribute to the immunosuppressive activity that renders IDO1 an interesting target of pharmacological inhibitors for cancer therapy [52].

A link between metabolic, inflammatory, and iron-regulatory pathways has been provided by evidence showing that mTOR regulates iron homeostasis by decreasing transferrin receptor (TfR1) stability and hence iron uptake. mTOR activates tristetraprolin (TTP), a protein involved in suppression of immune responses, which binds to TfR1 mRNA and enhances its degradation [53]. At the same time, TTP, which is induced by iron deficiency, promotes downregulation of iron-requiring genes, in particular those coding for mitochondrial proteins involved in energy production, thus facilitating survival when iron is scarce. Although these results were found in fibroblasts and myoblasts, it is tempting to speculate that a similar process can occur in macrophages, consistent with the demonstration that metabolic reprogramming mediated by the mTOR pathway is required for macrophage alternative activation [20].

Another example of the interaction between iron and immunometabolism was provided by the comparison of differences in gene expression profiles between mouse non-activated bone marrow-derived macrophages and non-activated peritoneal macrophages, which highlighted a differential expression of genes involved in heme biosynthesis [24], thus confirming a previous observation in human in vitro polarized macrophages [32].

## 6. Concluding Remarks

Recent advances in the availability of molecular and biochemical methods have highlighted that intracellular metabolic pathways control the effector functions of macrophages, as exemplified by the distinct metabolic signature of differently polarized macrophages, which show significant differences in pathways of energy production, amino acid metabolism and iron homeostasis. These processes are intertwined and we have tried to draw attention to the role of iron in these settings.

In particular, the effect of variations in iron availability on immunometabolism may impinge on the relevant role played by macrophages in several pathologic conditions like obesity, diabetes, cardiovascular diseases, tumors. Indeed, iron homeostasis is altered in adipose tissue associated macrophages, which have a pathogenetic role in obesity favoring insulin resistance [54], and macrophage iron is key in the development and progression of atherosclerotic plaques (discussed in Reference [55]).
